# Enhanced Charge Separation and FRET at Heterojunctions between Semiconductor Nanoparticles and Conducting Polymer Nanofibers for Efficient Solar Light Harvesting

**DOI:** 10.1038/srep17313

**Published:** 2015-11-27

**Authors:** Samim Sardar, Prasenjit Kar, Hynd Remita, Bo Liu, Peter Lemmens, Samir Kumar Pal, Srabanti Ghosh

**Affiliations:** 1 Department of Chemical, Biological and Macromolecular Sciences, S. N. Bose National Centre for Basic Sciences, Block JD, Sector III, Salt Lake, Kolkata 700 098, India; 2 CNRS, Laboratoire de Chimie Physique, UMR 8000, Université Paris-Sud, 91405 Orsay, France; 3 Laboratoire de Chimie Physique, UMR 8000-CNRS, Bât. 349, Université Paris-Sud, Université Paris Saclay, 91405 Orsay, France; 4 Institute for Condensed Matter Physics, TU Braunschweig, Mendelssohnstraβe 3, 38106 Braunschweig, Germany; 5 Laboratory for Emerging Nanometrology, TU Braunschweig, Braunschweig, Germany

## Abstract

Energy harvesting from solar light employing nanostructured materials offer an economic way to resolve energy and environmental issues. We have developed an efficient light harvesting heterostructure based on poly(diphenylbutadiyne) (PDPB) nanofibers and ZnO nanoparticles (NPs) *via* a solution phase synthetic route. ZnO NPs (~20 nm) were homogeneously loaded onto the PDPB nanofibers as evident from several analytical and spectroscopic techniques. The photoinduced electron transfer from PDPB nanofibers to ZnO NPs has been confirmed by steady state and picosecond-resolved photoluminescence studies. The co-sensitization for multiple photon harvesting (with different energies) at the heterojunction has been achieved *via* a systematic extension of conjugation from monomeric to polymeric diphenyl butadiyne moiety in the proximity of the ZnO NPs. On the other hand, energy transfer from the surface defects of ZnO NPs (~5 nm) to PDPB nanofibers through Förster Resonance Energy Transfer (FRET) confirms the close proximity with molecular resolution. The manifestation of efficient charge separation has been realized with ~5 fold increase in photocatalytic degradation of organic pollutants in comparison to polymer nanofibers counterpart under visible light irradiation. Our results provide a novel approach for the development of nanoheterojunctions for efficient light harvesting which will be helpful in designing future solar devices.

The development of novel functional materials to harvest and convert solar energy has been recognized as an important step to sustainable energy resources and to meet the rising energy demand^1^,^2^,^3^. In addition, demanding environmental pollution issues prompt for finding potential solutions via solar energy routes to clean up water and environmental pollutions. The key to the success of solar energy conversion is the development of high performance materials having a well matched photo absorption with the solar spectrum, an efficient photoexcited charge separation to prevent electron–hole recombination and an adequate energy of charges that carry out the desired chemical reactions (for example, photocatalysis)^4^,^5^,^6^,^7^. Although, oxide-based semiconductors, for example TiO_2_, serve as the benchmark photo responsive materials, they are limited with respect to performance, e.g. in the absorption of ultraviolet light due to their wide band gap and their low-efficiency in charge separation^8^,^9^. The rapid recombination of photoexcited electron-hole pairs has been recognized as a key factor behind the observed small photon conversion efficiency^3^,^10^,^11^. Therefore, a tremendous effort has been made to sensitize oxide based semiconductors in the visible spectral range *via* doping or surface-tuning^12^,^13^ Plasmonic photocatalysts have appeared as a very promising route to enhance solar light harvesting of TiO_2_^14^,^15^,^16^,^17^. Nevertheless, the high cost and low environmental stability of noble metal doped photocatalysts (i.e., Ag, Au) significantly limit their large-scale application. In fact, no single material can meet all stringent requirements as ideal solar energy converters. However, coupling of two materials as a semiconductor heterojunction has been demonstrated as an effective strategy allowing both a broader range of solar light absorption as well as a significant promotion of photo generated charge separation, thereby considerably improving their solar light harvesting efficiency^18^,^19^,^20^,^21^,^22^. Along this way, we have studied ultrafast photoinduced charge separation and charge recombination processes at the semiconductor-semiconductor (PbS-ZnO) interface for efficient solar light harvesting^23^.

Interestingly, the amalgamation of semiconductor nanocrystals and polymers is opening an efficient pathway for the development of multifunctional materials that demonstrate superior electrical, optical and mechanical properties^24^,^25^,^26^,^27^,^28^,^29^. Hence, incorporating semiconductor nanocrystals into conjugated polymers can complement the spectral absorption range of the polymers as well as allow to sensitize the semiconductor nanocrystals for renewable energy applications such as bulk heterojunction–type photovoltaics and photocatalysis^22^,^30^,^31^,^32^,^33^,^34^. A series of semiconductor nanocrystals and bulk conjugated polymer based heterojunctions such as TiO_2_-PANI, TiO_2_-P3HT has been studied for the harvesting of visible light^35^,^36^,^37^. The integration of another potential semiconductor nanocrystal, ZnO to the conjugated polymer has shown an improved efficiency under solar light^38^,^39^ ZnO nanoparticles (NPs) are believed to be nontoxic and biocompatible and have been used in many applications in our daily life, such as drug carriers and cosmetics etc^40^. Although semiconductor and conducting polymer heterojunctions demonstrate certain visible-light activity, the reported photo-conversion efficiency is small^39^. Furthermore, as the interfacial area is critical in achieving the favorable charge separation, it is essential to involve nanoscale units in creating the heterojunctions. Thereby a large interface area and reduced charge transport length towards the heterojunction may be achieved. Hence, designing heterostructures with the appropriate morphological orientation and band position of the semiconductor and the polymer unit is challenging. Conjugated polymer nanostructures are emerging materials for energy conversion and storage applications owing to their advantages of low cost facile synthesis, exceptional electrochemical activity and high carrier mobility^41^,^42^. The use of polymerizable organic moieties as sensitizers with a wide band gap semiconducting material has added the advantage of harvesting lower energy photons through the extension of conjugation to the polymeric form of the moiety. In this regard, we have recently reported the first experimental evidence of visible light responsive photocatalytic activity of conjugated polymer polydiphenylbutadiyne (PDPB) nanofibers for water depollution^43^. Moreover, we have shown that the polymer nanostructure is essential for the photoresponse in the visible spectral range^44^.

In this paper, we employ conjugated poly(diacetylene) based PDPB nanofibers with ZnO NPs for the formation of a unique nanoheterojunction to explore the effective charge separation from polymer to semiconductor nanocrystals. Notably, charge transfer or separation at the interface is a crucial aspect which determines the efficiency of light harvesting across the heterojunctions and consequently surface reactions. Nevertheless there is a common consensus that heterojunctions facilitate the migration of photoexcited electrons and holes across the interfaces in order to reduce charge recombination or enhance charge separation. However, very few direct experimental evidences or spectroscopic observations have been explored to establish the photoinduced charge transfer mechanism at the heterostructure interface^44^,^45^. Thus, investigations of the role of crystal defects, trapping sites and exciton-relaxation on the interfacial processes through spectroscopic studies towards the rational design of light harvesting nanoheterojunction (LHNH) are essential. By virtue of having intrinsic photoluminescence (PL) of ZnO from defect state emission as well as conducting polymer from different oligomeric and polymeric chains, ultrafast spectroscopic studies have been employed to investigate the interfacial charge/energy transfer dynamics. Furthermore, to ensure efficient charge separation, as well as to address how nanoheterojunctions between PDPB and ZnO are beneficial for solar light harvesting, photodecomposition of methyl orange as a model pollutant in water has been evaluated under visible light irradiation.

## Results and Discussion

**Figure 1:**
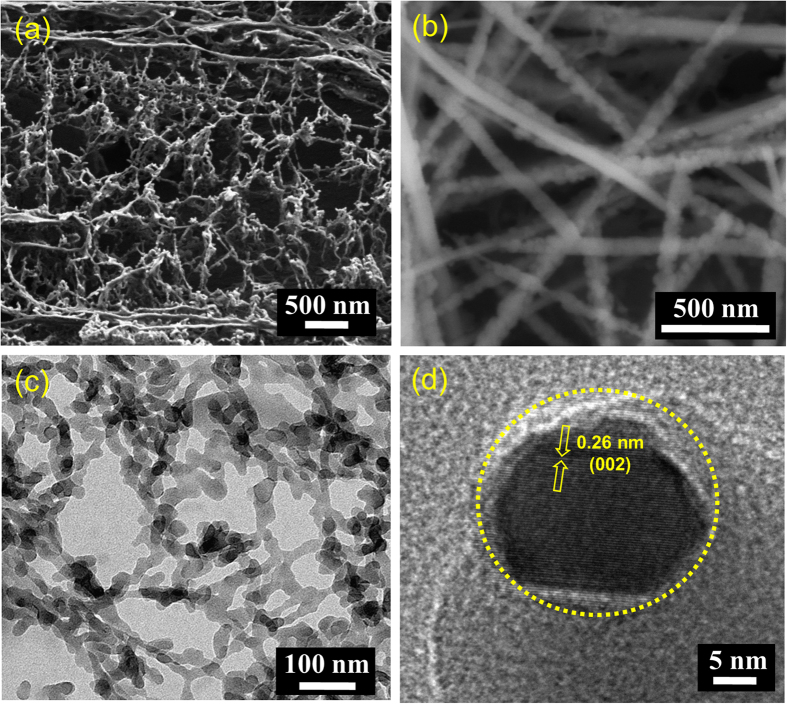
Microscopic images of PDPB nanofibers and PDPB-ZnO light harvesting nanoheterojunction (LHNH). SEM images of (**a**) PDPB nanofibers and (**b**) PDPB-ZnO LHNH.TEM images of (**c**) PDPB nanofibers and (**d**) PDPB-ZnO LHNH.

**Figure 2:**
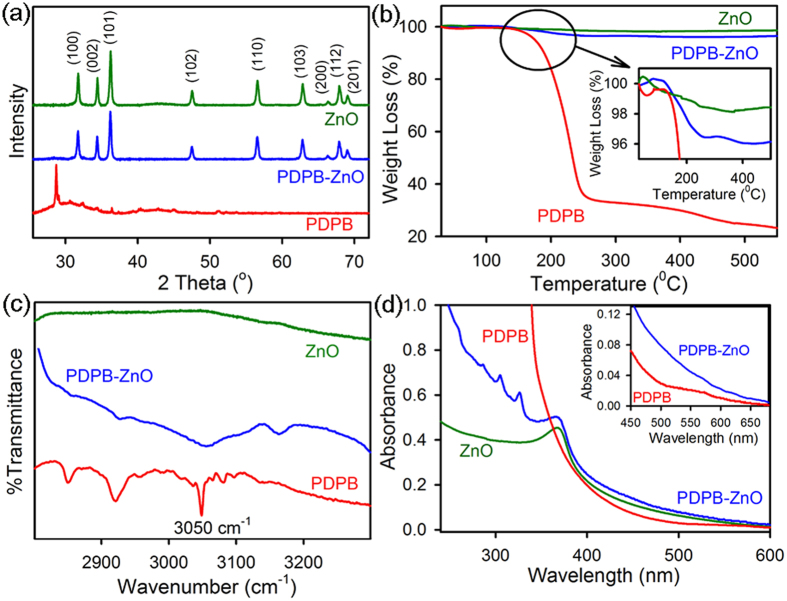
Characterization of PDPB nanofibers and PDPB-ZnO light harvesting nanoheterojunction. (**a**) X-ray diffraction patterns (**b**) thermogravimetric analysis profile (**c**) FTIR spectra and (**d**) UV-Vis absorption spectra of ZnO (green), PDPB-ZnO (blue) and PDPB (red).

For the fabrication of nanoheterojunctions, the first step is to synthesize conjugated polymer nanostructures. We have developed a soft template mediated controlled synthesis of PDPB nanofibers in hexagonal mesophases by UV irradiation^42^,^45^. The hydrophobic domain of the mesophases can accommodate high concentrations of 1, 4-diphenylbutadiyne (DPB) monomers, which can directly polymerize by photoirradiation in the presence of a free-radical initiator (benzoin methyl ether, BME)^46^. The DPB monomers undergo polymerization *via* 1, 4-addition reaction to form alternating ene-yne polymer chains upon irradiation with UV light. The PDPB-ZnO nanoheterojunctions were synthesized using ligand-free direct adsorption as well as *in situ* generation of ZnO NPs on polymer nanofibers by the coprecipitation method. A series of characterization techniques such as scanning electron microscope (SEM), transmission electron microscope (TEM), Fourier transform infrared spectroscopy (FTIR) and X-ray diffraction (XRD), UV–visible spectroscopy have been used to establish the formation of nanoheterojunctions. Figure 1 (a,b) illustrates the SEM images of PDPB nanofibers and PDPB-ZnO light harvesting nanoheterojunctions (LHNH), respectively. It can be seen that the polymer nanofibers have high-density with average diameter of 22 nm and few micron length. For PDPB-ZnO, Fig. 1b clearly shows the bright spots with the incorporation of 20 nm ZnO NPs, which cover the walls of PDPB nanofibers. Figure 1c illustrates a transmission electron microscopy image of PDPB nanofibers of uniform diameter of ~19 nm and a few micrometers long which are consistent with the SEM images. Notably, this one-dimensional structure reflects the geometry of the hydrophobic domains of the hexagonal mesophases. These polymer nanofibers are formed by π-stacking of PDPB oligomers^43^. A typical high-resolution TEM (HRTEM) image for PDPB-ZnO is shown in Fig. 1(d) which demonstrates the presence of PDPB and crystalline ZnO NPs (~20 nm) in which nanocrystals are embedded in the PDPB nanofibers. The ZnO NPs possess a good degree of cystallinity as discernible from the lattice fringes in HRTEM and the interplanar distances of ~0.26 nm corresponding to the spacing between two (002) planes of ZnO^47^. Furthermore, the crystallinity of ZnO NPs within the nanoheterojunction is corroborated by XRD. Figure 2a illustrates XRD patterns of pure PDPB nanofibers, PDPB-ZnO and ZnO NPs. A comparably small crystallinity is observed in the XRD pattern of PDPB nanofibers with a broad peak that may arise due to diffraction from polymeric chains. On the other hand, well-defined sharp peaks are observed at 2*θ* = 31.76°, 34.4°, 36.2°, 47.5°, 56.6°, 62.8°, 66.4°, 67.9°, and 69° which can be indexed as the (100), (002), (101), (102), (110), (103), (200), (112) and (201) diffraction planes of wurtzite structure (JCPDS card number: 36–1451), respectively, confirming the phase and crystallinity of ZnO NPs within the PDPB-ZnO LHNH. However, the broad peak originated from the PDPB nanofibers is not identified within the LHNH due to very low loading of polymer.

The loading of polymer as well as the thermal stability of the PDPB-ZnO has been studied by thermogravimetric analysis (TGA). Figure 2b shows the thermogravimetric curves of each material. The TGA graph of PDPB nanofibers showed onset of decomposition at about 185°C until a major decomposition happens around ~250 °C may be attributed to large-scale thermal degradation of polymeric chains. In contrast to polymer, pure ZnO NPs are very stable in air and almost no decomposition takes place in the range of 30–600 °C. The thermal decomposition of PDPB nanofibers in PDPB-ZnO LHNH is about 175 °C, which is slightly lower than that of pure PDPB (185 °C) may be associated with the interaction of inter-chains in PDPB macromolecule with ZnO NPs. The PDPB-ZnO LHNH exhibits a PDPB mass of as low as 4% determined from TGA curves (inset of Fig. 2b). As the carbon based polymers having high surface area with nanofibers morphology, it is expected to accommodate large number of high density ZnO NPs on the polymer surface as shown in Fig. 1b. Thus the weight percentage of polymer is lower compared to that of ZnO in the LHNH. This observation confirms the superlative formation of hybrid nanoheterojunction retaining the characteristic properties of each element. Additionally, chemical interaction between PDPB and ZnO has been monitored by Fourier transform infrared spectroscopy (FTIR) as shown in Fig. 2c. The characteristic peaks of pure PDPB at 3050 cm^−1^ corresponds to the C–H vibration involving hydrogen atoms in the para and meta positions. Pure ZnO did not show any characteristic peak in this spectral region. In contrast to the pure PDPB, the absorption peaks corresponding to C–H vibration get shifted to the high wave number and also become wider in PDPB-ZnO. This observation confirms that the ZnO NPs are well incorporated into the PDPB nanofibers.

UV–Visible absorption spectra of pure PDPB, PDPB-ZnO and ZnO nanostructures are presented in Fig. 2d. The PDPB nanofibers show a broad absorption in the visible range. The bare ZnO NPs demonstrate an absorption peak at 365 nm, corresponding to the band-gap excitation (E_g_ = 3.37 eV). The PDPB-ZnO spectrum has the characteristics of both PDPB and ZnO, and it exhibits enhanced absorption intensity in the visible range. This suggest that PDPB-ZnO can efficiently absorb visible light which is attributed to the PDPB absorption (see inset of Fig. 2d) and indicates that these heterojunctions could be useful in harvesting energy for optoelectronic applications.

**Figure 3:**
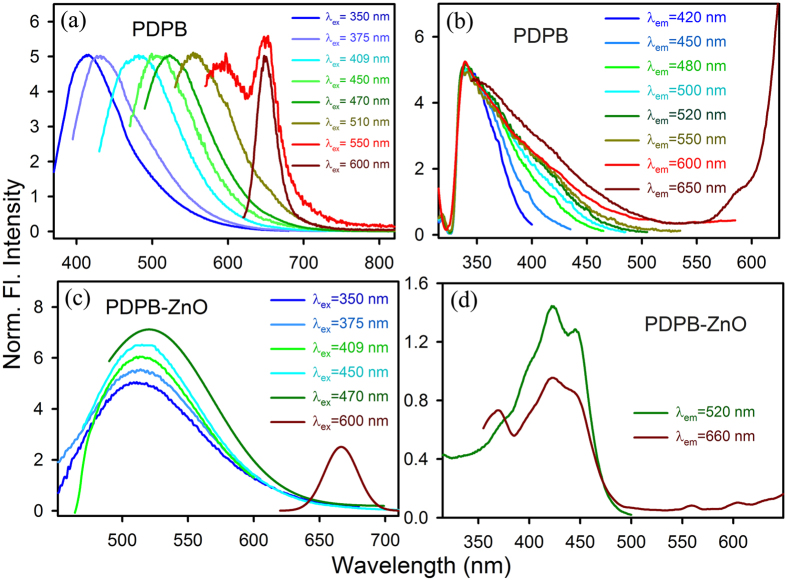
Steady state emission and excitation of PDPB nanofibers and PDPB-ZnO light harvesting nanoheterojunction. Room temperature PL spectra of (**a**) PDPB (**c**) PDPB-ZnO at different excitation wavelengths. The excitation spectra of (**b**) PDPB (**d**) PDPB-ZnO monitored at different emission maxima are shown. All spectra were recorded in ethanol.

In order to investigate the interfacial charge separation at the PDPB-ZnO nanoheterojunction which is the key step for efficient light harvesting, the photoinduced carrier dynamics is monitored by steady state and picosecond resolved fluorescence studies. Room temperature PL spectra of PDPB exhibit a strong dependency on the excitation wavelength as shown in Fig. 3a. The emission peak is red shifted from 410 nm to 650 nm at different excitation wavelengths from 350 nm to 600 nm. This suggests the presence of multiple emitting states in the polymer nanofibers which are associated with the wide extend of conjugation in the different segments having various oligomeric and polymeric chains. This kind of phenomenon is well documented in the field of conjugated oligomers and polymers^48^. From the cyclic voltammetry measurement, we have estimated the bandgap to be 1.81 eV which is remarkably narrow for a polymer^43^. Additionally, these results are consistent with the calculated value of the PDPB band gap, which is 1.95 eV, on the basis of density functional theory. The energy gaps (E_g_) for PDPB oligomers and polymeric chains having number of monomeric units from 1 to 8, decrease as the length of the polymer chains increases (E_g_ = 3.99 eV for monomer whereas E_g_ = 1.96 eV for octamer) facilitating the greater charge delocalization. Thus the multiple photoluminescence of PDPB can be attributed to the various oligomers present in the system. It has to be noted that the emission at 650 nm for the excitation wavelengths above 510 nm is originated from the conjugated polymer chain which acts as molecular wire. The multiple photoluminescence are further investigated from the excitation spectra as shown in Fig. 3b. The excitation spectra of PDPB at different detection wavelengths reveal a maximum at 340 nm which is consistent with the absorption spectra of PDPB as shown in Fig. 2d. As the detection wavelength shifts from 420 nm to 600 nm, the tail of the excitation spectra extends to the visible region due to the extended conjugation in PDPB nanofibers. Moreover, the excitation spectra monitored at a wavelength of 650 nm shows a peak around 590 nm which can be attributed to the long conjugated polymeric chain. In contrast, the emission spectrum of the PDPB-ZnO LHNH is independent on the excitation wavelength from 350 nm to 470 nm due to oligomeric units of the polymer, as shown in Fig. 3c. However, the emission spectrum of LHNH illustrates peak at 665 nm upon excitation at 600 nm, attributed to polymer chains. This observation clearly indicates the strong electronic interaction between semiconducting PDPB nanofibers and ZnO NPs. Due to the formation of nanoheterojunction with a large interfacial area the mobility of the excitons towards the interface is facilitated^44^. Consequently, the excited electrons from the polymer nanostructures instantly transfer to the ZnO NPs and eventually deexcite through defect centers located at the near surface that arise from oxygen vacancies^49^. The excitation spectrum of PDPB-ZnO as shown in Fig. 3d at the detection wavelength 520 nm reveals two distinct peaks at 420 nm and 445 nm which are consistent with the band gap absorption of the oligomers.

**Figure 4:**
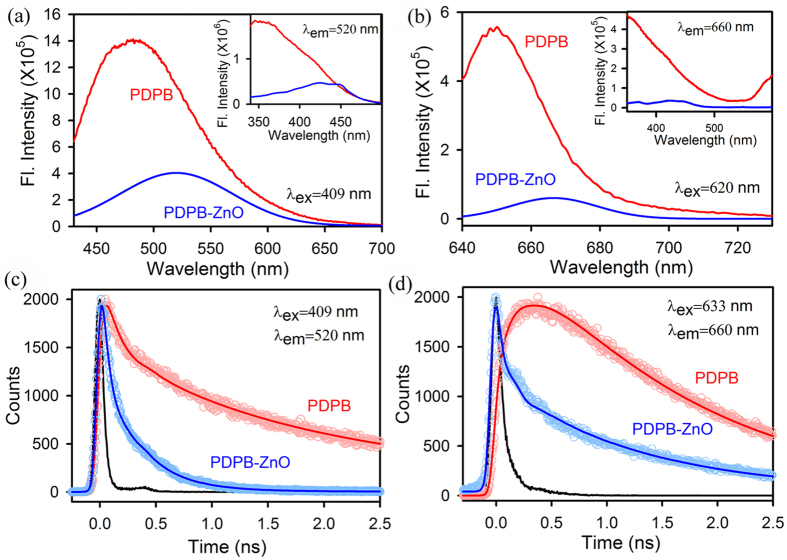
Steady state and time resolve spectroscopy of PDPB nanofibers and PDPB-ZnO light harvesting nanoheterojunction. PL spectra of PDPB, PDPB-ZnO at excitation wavelengths (**a**) 409 nm and (**b**) 633 nm. The inset shows excitation spectra monitored at 520 nm and 660 nm, respectively. Fluorescence decay profiles of PDPB and PDPB-ZnO at (**c**) 520 nm (excitation at 409 nm) (**d**) 660 nm (excitation at 633 nm).

The room temperature PL spectra of PDPB nanofibers show an emission peak at 485 nm upon excitation at 409 nm, as shown in Fig. 4a. The intensity of the emission peak decreases considerably and is red shifted to 520 nm when the polymers are attached to the ZnO NPs. The shift is due to the strong electronic interaction and energy level alignment at the nanoheterojunction. The inset of Fig. 4a shows the corresponding excitation spectra. The picosecond resolved fluorescence decays (Fig. 4c) of PDPB and PDPB-ZnO LHNH were measured upon excitation with 409 nm laser, and monitored at a wavelength of 520 nm. In case of PDPB-ZnO LHNH, the decay curve of PDPB shows significant shorter lifetime of 30 ps (74%) as compared to that of PDPB; 140 ps (45%) (Table 1). The observed decrease in lifetime can be correlated to the electron transfer process from PDPB oligomers to the ZnO NPs. The charge separation from polymeric chain of PDPB to ZnO NPs is also monitored from steady state and time resolved spectroscopy, as shown in Fig. 4b,d, respectively. The steady state emission peak decreases and is red shifted to 665 nm for PDPB-ZnO LHNH upon excitation at 620 nm. The inset of Fig. 4b shows the corresponding excitation spectra. As shown in Fig. 4d, the fluorescence decay curve for PDPB upon excitation at 633 nm shows an intrinsic buildup with a rise component of 290 ps (monitored at 660 nm) due to delocalization of electron in conjugated polymeric chain. The emission decay curve of PDPB is fitted with a rise followed by single exponential decay function with a lifetime of 1.58 ns. However, the decay curve of PDPB-ZnO LHNH deviates from single exponential to biexponential showing one significant shorter lifetime 30 ps (87%) and a longer lifetime of 1.24 ns (13%). Hence, the efficient photoinduced charge separation takes place at the nanoheterojunction where electrons are transferred from the conjugated polymer nanofibers to the ZnO NPs and holes remain in the polymers.

After studying the interfacial dynamics at the nanoheterojunction using ZnO NPs with approximate size ~20 nm which does not have intrinsic defect state emission, ZnO NPs (~5 nm) were synthesized *in situ* on PDPB nanofibers to investigate the role of defect states in the photoinduced charge transfer processes. Interestingly, during *in situ* synthesis of ZnO NPs on PDPB nanofibers, the average grain size of the ZnO is 5 nm which is similar to the size of ZnO NPs if formed alone. This suggests that the growth of ZnO NPs is not affected by the presence of PDPB nanofibers as well as the formation of the nanoheterojunctions is successful.

**Figure 5:**
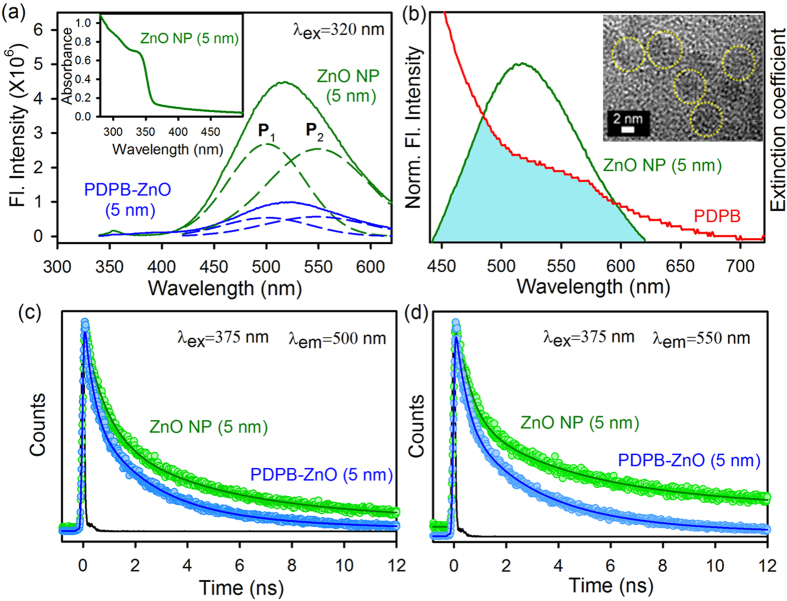
FRET study between PDPB nanofibers and ZnO nanoparticles (5 nm). (**a**) Room temperature PL spectra of ZnO NPs (green) and PDPB-ZnO LHNH (blue) are shown. The excitation wavelength was at 320 nm. The broad emission band is composed of two components, P_1_ (500 nm) and P_2_ (555 nm). Inset shows the absorption spectrum of ZnO NPs (~5 nm). (**b**) Shows the overlap of the ZnO NP emission and PDPB absorption. Inset shows the HRTEM image of *in situ* synthesized ZnO NPs (~5 nm) on PDPB nanofibers. The picosecond-resolved fluorescence transients of ZnO NPs (excitation at 375 nm) in the absence (green) and in the presence of PDPB (blue) collected at (**c**) 500 nm and (**d**) 555 nm are shown.

The ZnO NP (~5 nm) has an intrinsic defect state emission as shown in Fig. 5a. Inset of Fig. 5a illustrated the absorption spectrum of the corresponding ZnO NPs. The room temperature PL spectrum of ZnO NP is composed of two emission bands upon excitation above the band-edge (λ_ex_ = 320 nm). The narrow band at 360 nm in the emission spectra is due to the band gap emission. The broad emission band in the blue green region is associated with surface defect centers which is comprised of two bands: one arises from the doubly charged vacancy center (V_0_^++^) located at 550 nm (P_2_) and the other one arises from the singly charged vacancy center (V_0_^+^) located at 500 nm (P_1_)^49^,^50^,^51^. The emission intensity decreases when ZnO NPs are attached to the PDPB nanofibers (inset Fig. 5b showed TEM image of PDPB-ZnO, 5 nm) which can be attributed to photoinduced non-radiative processes from ZnO NPs to the PDPB nanofibers. There is significant spectral overlap between ZnO NP emission and PDPB absorption as shown in Fig. 5b which leads to a probable energy transfer. Therefore, we propose Förster resonance energy transfer (FRET) from the donor ZnO NPs to the acceptor PDPB nanofibers^50^. The picosecond resolved fluorescence decay profile of the donor ZnO NPs in the presence and absence of the acceptor PDPB were obtained upon excitation with a 375 nm laser and monitored at 500 nm (P_1_) and 550 nm (P_2_) (Fig. 5c,d, respectively). A shorter excited state lifetime of the ZnO NPs is clearly observed in the presence of PDPB. Details of the spectroscopic fitting parameters of the fluorescence transients are tabulated in Table 2. From FRET calculations, the distance between the donor ZnO NPs and acceptor PDPB nanofibers are determined to be 3.4 nm and 3.1 nm for P_1_ and P_2_ states, respectively. The FRET distance is consistent with the size of the ZnO NPs (radius = 2.5 nm). The energy transfer efficiency is calculated to be 64% and 70% from P_1_ and P_2_ states, respectively. This observation confirms the proximity between the PDPB and ZnO as well as UV light harvesting in the LHNH via energy transfer from ZnO to PDPB nanofibers.

**Figure 6:**
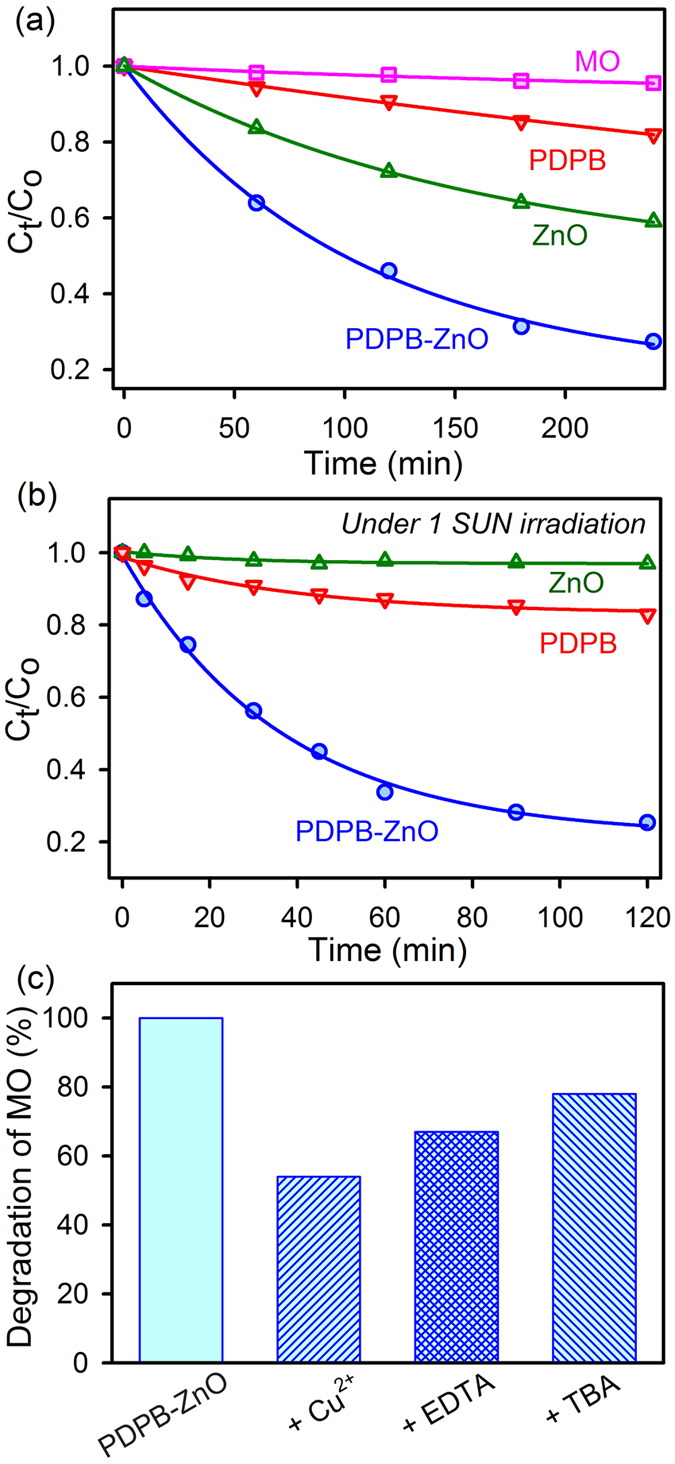
Photocatalytic activity of PDPB nanofibers, ZnO nanoparticles and PDPB-ZnO light harvesting nanoheterojunction. Photocatalytic degradation of MO in the presence of PDPB (red), ZnO (green) and PDPB-ZnO (blue) under (**a**) UV light (**b**) Visible light (1 sun) irradiation. (**c**) Effect of Cu^2+^, EDTA and TBA on the photocatalytic activity of PDPB-ZnO LHNH. The photodegradation reaction of MO (initial concentration C_0_ = 0.3 × 10^−4^ M) was carried out in a 10 mm optical path quartz cell reactor containing 3.5 mL of a model solution with a concentration of 1 g.L^−1^ of the PDPB-ZnO, PDPB nanofiber (0.2 g.L^−1^) and ZnO NPs (1 g.L^−1^).

In order to investigate the manifestation of the interfacial charge transfer dynamics in PDPB-ZnO heterojunction, the photocatalytic activity has been studied using methyl orange (MO) as a model pollutant. Photocatalysis involves charge carrier generation and mobility from oxide based semiconductor nanomaterials and recognized as a green technology for the purification of water. Adsorption tests under dark condition showed that MO does not adsorb on the surface of the PDPB-ZnO LHNH. The photocatalytic activity of the LHNH for degradation of MO under UV-VIS and Visible light was compared with ZnO NPs and PDPB nanofibers as a control experiment (Fig. 6a,b). Figure 6a demonstrates the efficient photocatalytic activity of PDPB-ZnO LHNH (74%) in comparison to both counter parts (PDPB, 18% and ZnO, 40%) under 240 min UV-VIS light irradiation (λ ≥ 365 nm). Figure 6b shows that the PDPB-ZnO LHNH is also very active for photocatalytic degradation of MO under 120 min visible light irradiation. However, ZnO NPs having wide a band gap did not show any visible light activity. The activity of the polymer nanostructures is much lower ~17% in comparison to PDPB-ZnO LHNH (80%) under similar illumination conditions. These observations suggest that due to efficient charge separation at the interface between PDPB and ZnO, the PDPB-ZnO LHNH is suitable for solar light harvesting. This novel photocatalyst exhibits an additional advantage compared to unsupported ZnO or TiO_2_ photocatalysts, it does not require any filtration step as it settles down quickly after use in the photocatalytic reactor. The ease of solution based synthesis and good photocatalytic activity of PDPB-ZnO LHNH make them promising nanostructures for environmental applications. During photocatalytic reaction, photoinduced electrons and holes escape recombination and migrate to the semiconductor surface which consequently generates (in the presence of oxygen and water) highly oxidative radicals, that can degrade the organic pollutants. A large number of reactive species including h^+^, ^∙^OH, and O_2_^∙−^ are involved in the photocatalytic oxidation process^52^. Hence, the effects of free radical scavengers on the degradation of MO were examined to elucidate the reaction mechanism. To investigate the role of the excess electrons, Cu^2+^ was used as a scavenger (it reacts with electron to yield Cu^+^)^52^. Tertiary butyl alcohol (TBA) was introduced as the scavenger of ^∙^OH, and ethylenediaminetetraacetic acid (EDTA) was adopted to quench the holes (h^+^)^53^. As a consequence of quenching, the photocatalytic oxidation reaction is partly suppressed. The effects of a series of scavengers on the degradation efficiency of MO are shown in Fig. 6c. The degradation efficiency of PDPB-ZnO photocatalyst for MO is reduced to 44% after adding Cu^2+^. A similar trend is observed after the addition of EDTA and TBA. The corresponding photodegradation efficiencies decreased to 49% and 55%, respectively. According to the above experimental results, it can be clearly seen that O_2_^∙−^ and h^+^ are the main reactive species in the photocatalytic oxidation process of MO, whereas ^∙^OH has a minimal effect on this process.

**Figure 7:**
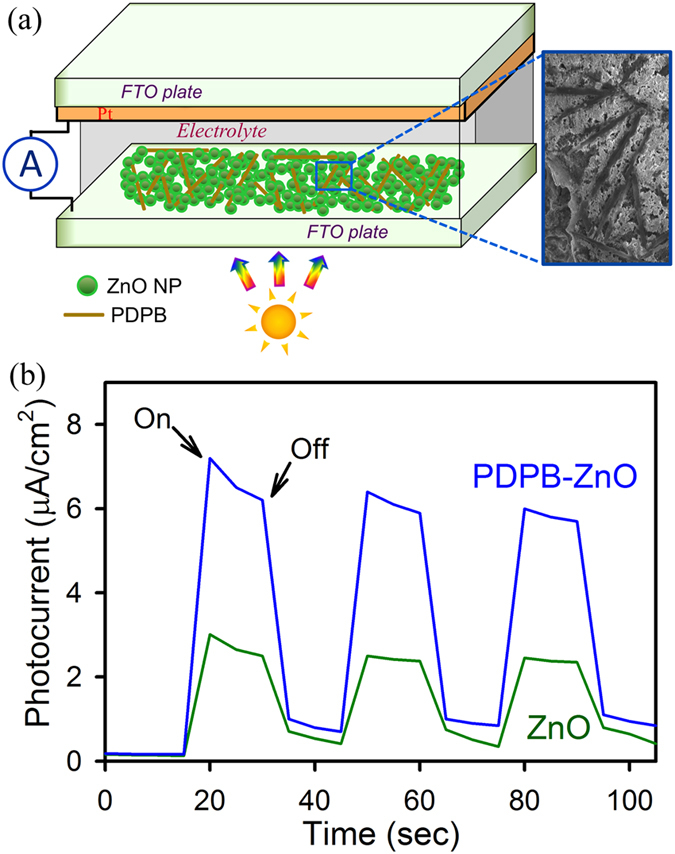
Photocurrent response of ZnO nanoparticles and PDPB-ZnO light harvesting nanoheterojunction. (**a**) Schematic representation of photocurrent measurement set up using dye-sensitized solar cell geometry. (**b**) Photocurrent responses of PDPB-ZnO LHNH and ZnO NPs without any bias voltage under 1000 W m^−2^ incident power irradiation from a light source.

**Figure 8:**
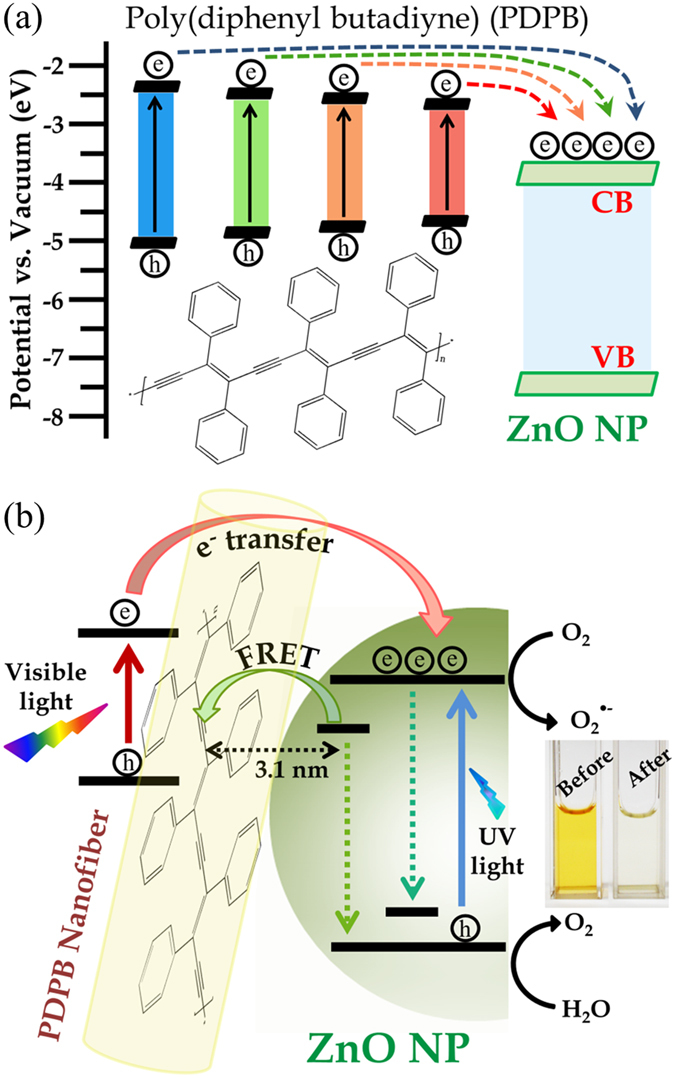
Schematic presentation of (**a**) the co-sensitization of different PDPB oligomers to ZnO NPs and molecular structure of PDPB polymer (**b**) the interfacial carrier dynamics at the heterojunction showing the photocatalytic degradation of MO in aqueous solution.

A direct evidence of enhanced charge separation at the nanoheterojunction interface is followed by photocurrent measurements. Photocurrent generation of ZnO and PDPB-ZnO films deposited onto ITO electrodes were studied under white light irradiation (at 1000 W/m^2^) in solar cell geometries, as shown in Fig. 7a. A steady and rapid cathodic photocurrent response is obtained from the LHNH film when the irradiations is switched on and off, see Fig. 7b. For PDPB-ZnO, the photocurrent response was 7.2 μA/cm^2^, about 2.5 times higher than pure ZnO NPs which reflects higher separation and transfer efficiency of photo excited electrons from conjugated polymer to the conduction band of ZnO due to the formation of the heterostructure. The PDPB is a p-type, organic semiconductor; ZnO is an inorganic, n-type semiconductor and forming a donor-acceptor junction (heterojunction). When the PDPB-ZnO LHNH is illuminated under visible light, electrons are excited from the HOMO to the LUMO of PDPB, leaving holes behind in the HOMO of PDPB. The excited state electrons are readily injected into the conduction band of ZnO as schematically presented in Fig. 8a. Consequently, we have observed enhanced photocatalytic activity and photoresponse due to formation of the nanoheterojunction, as shown in Fig. 8b. In future, we will consider the optimization of the cell architecture, utilization of other types of semiconductors and conducting polymer nanostructures to obtain a significantly enhanced solar light harvesting.

## Conclusion

In summary, we have successfully demonstrated that a simple adsorption of ZnO NPs on PDPB nanofiber surface leads to the effective sensitization of semiconductor nanocrystals for solar light harvesting. The formation of PDPB-ZnO LHNH has been confirmed by several microscopic and analytical techniques. Appropriate band gap alignment in combination with easy solution-processability, highlights this material as a promising candidate for energy harvesting applications. The interfacial carrier dynamics which is the key for efficient light harvesting has been unraveled by detailed steady state and ultrafast spectroscopic studies, suggesting the co-sensitization of ZnO NPs by multiple states of polymer nanofibers originated from oligomeric and polymer chain unit. The efficient charge separation at the interface leads to enhanced photocatalytic activity and photoresponse. Our approach provides simple and economic ways to design hybrid nanoheterojunction with several functionalities which are suitable for solar cell, chemical sensors and optoelectronic device applications.

## Methods

### Reagents

Sodium dodecyl sulfate (SDS), sodium chloride, cyclohexane (>99%), pentanol (≥99%), zinc acetate dihydrate, ZnO (~30 nm), platinum chloride, (H_2_PtCl_6_) and methyl orange were purchased from Sigma-Aldrich. For *in-situ* polymerization, we used 1, 4-diphenylbutadiyne (DPB) (Aldrich) as monomer and benzoin methyl ether (BME) (Fluka) as catalyst. All compounds were used as received. Ultrapure water (Millipore System, 18.2 MΩ cm) and ethanol (≥99% for HPLC, purchased from Sigma-Aldrich) were used as solvents. Analytical grade chemicals were used for synthesis without further purifications. Ethylenediaminetetraacetic acid (EDTA), sodium hydroxide (NaOH) and tertiary butyl alcohol (TBA) was purchased from Merck.

### Synthesis of polymer nanostructures in mesophases

The swollen hexagonal mesophases were prepared following the previously published method with some modifications^42^,^45^. Typically, 1 g of the surfactant (Sodium Dodecyl Sulfate) was dissolved in 2 mL of 0.3 mol.L^−1^ NaCl in glass tubes. After a vigorous stirring at 30 °C, the surfactant had completely dissolved leading to a transparent and viscous micellar solution. The subsequent addition of cyclohexane containing monomer 1, 4-diphenylbutadiyne (DPB) (10% of mass) and initiator benzoin methyl ether (BME) (1%) in the micellar solution under stirring leads to a white unstable emulsion. A cosurfactant, pentanol-1 (420 μL), was then added to the mixture, which was then strongly stirred for a few minutes. This led to a perfectly colorless, translucent, birefringent and stable gel, a hexagonal mesophase. The doped mesophases were used as soft templates to synthesize polymer nanostructures induced by irradiation using UV light with an Oriel 300 W Xenon UV-visible lamp at a distance of 5 cm for 12 hours. After reaction, the materials were extracted in a water-ethanol mixture, centrifuged, and washed several times to eliminate the surfactant.

### Preparation of PDPB-ZnO nanoheterojunction

PDPB–ZnO nanoheterojunction were prepared by dispersing ZnO (20 nm) into the ethanolic solution of as prepared PDPB nanofibers. The solutions were stirred for 48 hours in the dark and then separated by centrifugation. The transparent supernatant was removed and the remaining yellowish powder was re-dispersed in ethanol for characterization. The LHNH was then dried in a water bath and stored in the dark until further use. During the synthesis of PDPB–ZnO nanoheterojunction, we used 5%, 10%, 20% and 50% of PDPB nanofibers with respect to ZnO nanoparticles (20 nm). We choose the lower concentration (5%) of PDPB nanofibers for further detailed study. The photocatalytic activity of PDPB–ZnO nanoheterojunction is found to be independent of the concentration of PDPB nanofibers (data not shown). The ~20 nm ZnO NPs does not have intrinsic defect state emission which is consistent with the literature. Hence, ZnO NPs (~5 nm) having intrinsic defect state emission have been employed for FRET study in order to confirm the molecular proximity between PDPB nanofibers and ZnO NPs within the LHNH.

For the direct *in situ* synthesis of ZnO NPs on PDPB nanofibers, we adopted a coprecipitation technique using ethanol as the solvent, following the previous reports from our group^50^,^54^,^55^. Briefly, 20 mL of 4 mM zinc acetate dehydrate solution was heated at 70 °C for 30 min in the presence of a fixed amount of PDPB nanofibers (1 mg). Then 20 mL of 4 mM sodium hydroxide solution in ethanol was then added and the mixture was hydrolyzed for 2 h at 60 °C to obtain NPs of average diameters of ~5 nm.

### Characterization methods

Field Emission Scanning Electron Microscopy (FESEM, QUANTA FEG 250) was used to investigate the surface morphology of the samples. Transmission electron microscopy (TEM) was carried out using an FEI (Technai S-Twin) instrument with acceleration voltage of 200 kV. A drop of sample was placed on a carbon-coated copper grid and particle sizes were determined from micrographs recorded at a high magnification of 100000X. X-ray diffraction (XRD) was used to characterize crystal phase by a PANalytical XPERTPRO diffractometer equipped with Cu Kα radiation (at 40 mA and 40 kV) at a scanning rate of 0.02° S^−1^ in the 2θ range from 20° to 80°. Fourier transform infrared spectroscopy (FTIR) was carried out with JASCO FTIR-6300 spectrometer by the KBr Pellet method. Thermal gravimetric analysis (TGA) of PDPB, PDPB-ZnO and ZnO solid powder was performed under nitrogen atmosphere with a heating rate of 10 °C-min from 30 °C to 600 °C by using a Perkin-Elmer TGA-50H. For optical experiments, the steady-state absorption and emission were recorded with a Shimadzu UV-2600 spectrophotometer and a Jobin Yvon Fluoromax-3 fluorimeter, respectively. Picosecond-resolved spectroscopic studies were carried out using a commercial time correlated single photon counting (TCSPC) setup from Edinburgh Instruments (instrument response function, IRF = 80 ps, excitation at 375 nm, 409 nm and 633 nm). The details of experimental set up and methodology were described in our earlier report^56^,^57^. The average lifetime (amplitude-weighted) of a multi-exponential decay is expressed as 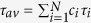. The Förster Resonance Energy Transfer (FRET) has been studied between donor (ZnO) and acceptor (PDPB) by following traditional methodology^58^ by calculating Förster distance (R_0_ in Å)


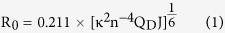


where, κ^2^ is a factor describing the relative orientation in space of the transition dipoles of the donor and acceptor and the magnitude is assumed to be 2/3. The refractive index (n) of the medium is considered to be 1.496. Q_D_, the integrated quantum yield of the donor in the absence of acceptor is measured to be 3.8 × 10^−3^. J, the overlap integral, which expresses the degree of spectral overlap between the donor emission and the acceptor absorption, is given by,


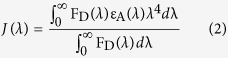


where, F_D_(λ) is the fluorescence intensity of the donor in the wavelength range of λ to λ + dλ and is dimensionless; ε_A_(λ) is the extinction coefficient (in M^−1^ cm^−1^) of the acceptor at λ. If λ is in nm, then J is in units of M^−1^cm^−1^nm^4^. The estimated value of the overlap integral is 2.77 × 10^13^. The donor-acceptor distance (r_DA_) can be easily calculated using the formula,


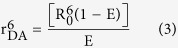


Here E is the efficiency of energy transfer. The transfer efficiency is measured using the relative fluorescence lifetime of the donor, in absence (τ_D_) and presence (τ_DA_) of the acceptor.


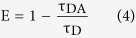


From the average lifetime calculation for the PDPB-ZnO LHNH, we obtain the effective distance between the donor and the acceptor (r_DA_), using the above equations.

### Photocatalytic tests

The photocatalytic activity of the PDPB-ZnO under visible light illumination has been tested for photodecomposition of methyl orange (MO), taken as model pollutant) in water. It is a representative of hazardous azo dyes. The photodegradation reaction of MO (initial concentration C_0_ = 0.3 × 10^−4^ M) was carried out in a 10 mm optical path quartz cell reactor containing 3.5 mL of a model solution with a concentration of 1 g.L^−1^ of the PDPB-ZnO. For control experiment, the PDPB nanofiber (0.2 g.L^−1^) and ZnO NPs (1 g.L^−1^) have been studied separately. The suspension was irradiated with a mercury lamp, λ ≥ 365 nm (under UV-visible light) and appropriate amount of aliquots were collected from the reactor at successive time intervals. The percentage degradation (%DE) of MO was determined using equation 5:


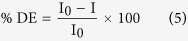


where, I_0_ is the initial absorption intensity of MO at λ_max_ = 460 nm and I is the absorption intensity after UV irradiation.

### Photocurrent measurements

Photocurrent measurements were done in a dye-sensitized solar cell (DSSC) setup^59^. For the fabrication of DSSCs, platinum NPs deposited on FTO substrates were used as counter electrodes. The platinum (Pt) NPs were deposited on the FTO substrates by thermal decomposition of 5 mM platinum chloride, H_2_PtCl_6_ solution in isopropanol at 385 °C for 30 min. PDPB-ZnO were used as the photoelectrode and the two electrodes were placed on top of each other with a single layer of 60 μm thick Surlyn (Solaronix) as a spacer between the two electrodes. A liquid electrolyte (1 M KCl) was used as the hole conductor and filled in the inter-electrode space by using capillary force, through two small holes (diameter = 1 mm) pre-drilled on the counter electrode. Finally, the two holes were sealed by using another piece of Surlyn to prevent the leakage of the electrolyte. In all of our experiments, the active area of the DSSC was fixed at 1 cm^2^.

## Additional Information

**How to cite this article**: Sardar, S. *et al.* Enhanced Charge Separation and FRET at Heterojunctions between Semiconductor Nanoparticles and Conducting Polymer Nanofibers for Efficient Solar Light Harvesting. *Sci. Rep.* **5**, 17313; doi: 10.1038/srep17313 (2015).

## Figures and Tables

**Table 1:** Dynamics of picosecond-resolved luminescence transients of PDPB and PDPB-ZnO LHNH^a^.

Sample	Excitation wavelength (nm)	Detection wavelength (nm)	*τ*_1_(ns)	*τ*_2_ (ns)	*τ*_3_(ns)
PDPB	409	520	0.14 (45.4%)	1.40 (34.8%)	4.85 (19.8%)
PDPB-ZnO	409	520	0.03 (74.2%)	0.30 (23.8%)	2.21 (2.0%)
PDPB	633	650	0.29 (−21%)	1.58 (121%)	
PDPB-ZnO	633	650	0.03 (86.7%)	1.24 (13.3%)	

^a^Numbers in parenthesis indicate relative weightages.

**Table 2:** Dynamics of picosecond-resolved luminescence transients of PDPB and PDPB-ZnO LHNH^b^.

Sample	Excitation wavelength (nm)	Detection wavelength (nm)	τ_1_(ns)	τ_2_ (ns)	τ_3_ (ns)	τ_avg_ (ns)
ZnO NP (5 nm)	375	500	0.82 (51.8%)	4.22 (40.7%)	29.30 (7.5%)	4.34
PDPB-ZnO	375	500	0.27 (53.3%)	2.15 (39.5%)	7.8 (7.2%)	1.55
ZnO NP (5 nm)	375	550	0.52 (51.3%)	4.01 (35.0%)	35.12 (13.7%)	6.48
PDPB-ZnO	375	550	0.34 (53.2%)	2.63 (39.8%)	9.89 (7%)	1.91

^b^Numbers in the parenthesis indicate relative weightages.
